# Relative Stabilities of Conserved and Non-Conserved Structures in the OB-Fold Superfamily

**DOI:** 10.3390/ijms10052412

**Published:** 2009-05-22

**Authors:** Kaitlyn M. Guardino, Sarah R. Sheftic, Robert E. Slattery, Andrei T. Alexandrescu

**Affiliations:** Department of Molecular and Cell Biology, University of Connecticut, Storrs, CT 06269, USA

**Keywords:** protein folding, structural genomics, structure similarity, protein dynamics, modularity

## Abstract

The OB-fold is a diverse structure superfamily based on a β-barrel motif that is often supplemented with additional non-conserved secondary structures. Previous deletion mutagenesis and NMR hydrogen exchange studies of three OB-fold proteins showed that the structural stabilities of sites within the conserved β-barrels were larger than sites in non-conserved segments. In this work we examined a database of 80 representative domain structures currently classified as OB-folds, to establish the basis of this effect. Residue-specific values were obtained for the number of Cα-Cα distance contacts, sequence hydrophobicities, crystallographic B-factors, and theoretical B-factors calculated from a Gaussian Network Model. All four parameters point to a larger average flexibility for the non-conserved structures compared to the conserved β-barrels. The theoretical B-factors and contact densities show the highest sensitivity. Our results suggest a model of protein structure evolution in which novel structural features develop at the periphery of conserved motifs. Core residues are more resistant to structural changes during evolution since their substitution would disrupt a larger number of interactions. Similar factors are likely to account for the differences in stability to unfolding between conserved and non-conserved structures.

## 1. Introduction

The three-dimensional structures of proteins underlie their functions. In spite of the growing number of structures available, the processes by which proteins fold remain poorly understood. What constitutes a protein fold – is a fold a distinct entity representative of a group of structures, or part of a continuum of similarity interrelationships [1–5]? Do recurring structure motifs have physical properties that would make them particularly suitable as folding nuclei [6,7] or are they modules propagated by chance throughout a variety of proteins [8]? What do recurrent structure motifs tell us about how protein structures evolve? Although many proteins fold in a cooperative two-state transition, other proteins are less cooperatively organized, making it possible to characterize partially folded intermediate states. These partially folded states may offer the best chance to understand protein-folding mechanisms [1,6,9–12]. Here we try to shed light on the evolutionary origins of partially folded states of OB-fold proteins.

**Figure 1.**
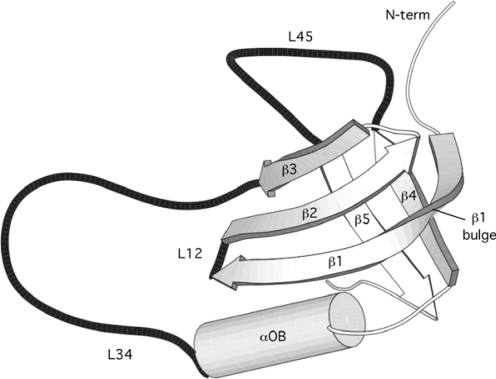
Prototypical OB-fold, illustrated with the NMR structure of SN-OB [6].

The OB-fold is a common structural motif originally identified as an Oligo-nucleotide/oligosaccharide-Binding fold [2]. As the number of protein structures with OB-fold motifs has grown, the superfamily has come to include proteins with considerably different functions; including metal-binding, protease inhibition, and chemotaxis. The OB-fold motif (Figure 1) consists of a 5-stranded anti-parallel Greek Key β-barrel, formed by a β-meander (strands 1 to 3) and a β-hairpin (strands 4 and 5). Strand 1 has a conserved β-bulge that allows anti-parallel connections of its N-terminus with strand 4 and C-terminus with strand 2. An irregular parallel pairing of strands 3 and 5 closes the barrel (Figure 1). Loops L12, L34, and L45 often contribute residues to substrate-recognition sites [2], which typically occur on the face of the β-barrel formed by the β1–3 meander [2,13–15]. A three-layer core of hydrophobic residues stabilizes the interior of the β-barrel [2]. In its original conception, the OB-fold motif included an α-helix between β-strands 3 and 4 [2]. This αOB element is much more weakly conserved than the β-barrel and in many OB-fold proteins is missing entirely. When present, the length of this α-helix and its orientation relative to the β-barrel shows large variation between different members of the OB-fold superfamily [6].

Aside from relatively minor variation in the conserved β-barrel framework, many OB-fold proteins contain additional non-conserved structures [7]. These accessory structures often impart unique activities. In the case of staphylococcal nuclease for example, part of the active site of the enzyme comes from a loop between two α-helices that are outside the OB-fold [6]. We first established using native state hydrogen exchange (HX) [7,16,17] NMR experiments and mutagenesis [6,16] that in the three OB-fold proteins CspA, LysN and SN, the conserved β-barrel motifs are more stable to unfolding than the non-conserved structures. The three proteins lack detectable sequence homology (sequence identities are below 10%). We noted a correlation between the site-specific stability data obtained from HX experiments, sequence parameters such as amino acid hydrophobicity [17] and structure parameters such as the number of distance contacts per residue [7]. These types of correlations have been observed with other types of globular proteins [18–20] and led us to question whether conserved motifs might have structure and sequence signatures that directly distinguish them from their non-conserved counterparts.

Here we extended the work by comparing the properties of conserved and non-conserved secondary structures within the entire superfamily of OB-fold proteins. We looked at 80 representatives of the 95 domain structures classified as OB-folds in the September 2007 version of the SCOP database [3,21], after excluding 16 domains that either had poor quality structures or a doubtful assignment to the OB-fold (please see details in the Experimental Section). We analyzed the remaining 80 domains in terms of four parameters: sequence hydrophobicity, number of distance contacts per residue, crystallographic B-factors, and theoretical B-factors calculated from a Gaussian Network Model (GNM) [22–24]. We found that on average the conserved β-barrels have more hydrophobic sequences, larger numbers of contacts per residue, and smaller experimental and predicted B-factors compared to the non-conserved secondary structures. The results suggest that structural conservation and microsopic stability are manifestations of the same physical forces that resist changes to the hydrophobic cores of protein structures.

## 2. Results

### 2.1. Length distribution of OB-fold secondary structures

**Figure 2.**
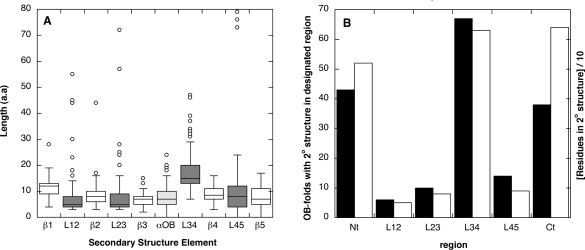
Distributions of regular and irregular structure in OB-fold proteins. (a) Box plots summarizing the length distributions of OB-fold β-strands, intervening loops, and the αOB helix. (b) Bar graph showing the number of domains out of 80 (black bars), that have an element of regular secondary structure within the indicated region. Also shown, the number of residues that participate in secondary structures within the specified regions divided by 10 (white bars). The graph shows that the longer loops accommodate nonconserved secondary structures more frequently, and tolerate longer secondary structures. Data are also included for the N- and C-termini (Nt and Ct, respectively), which unlike the loops have one free end.

The length distributions of secondary structures and intervening loops in the OB-fold motifs are summarized in the box plot shown in Figure 2A. The average lengths of the strands and loops are about eight residues, typical of the approximately 10-residue average length of secondary structure segments [25]. The length distributions are bell-shaped (boxes) with decaying tails of outliers that correspond to longer secondary structures (circles).

Notable exceptions to the trends are the loop L34 and to a lesser extent the first β-strand. Loop L34 is significantly longer (ρ < 0.001). Its length is probably a consequence of the need to traverse the distance from the C-terminus of the β1–3 meander to the N-terminus of the β4–5 hairpin on the other side of the β-barrel structure (Figure 1).

The other exception, strand β1, has a significantly larger mean length than the other strands. The N-terminus of β1 pairs with β4 and the C-terminus with β2. To satisfy pairing with both strands, β1 needs a conserved bend (Figure 1). Typically this bend is satisfied by a one-residue β-bulge [2] but in some cases more extensive perturbations, or even irregular linkers are found in this region.

We looked at pair-wise β-strand length correlations in the representative domains. The lengths of most strands were significantly correlated (ρ ≤ 0.008). As expected, the lengths of strands connected within the β-sheet (e.g. β1-β2, β4-β5) are similar to satisfy hydrogen-bonded pairing. The exceptions of strands with uncorrelated lengths were β2-β4, β2-β5, and β3-β4. These strands come from separate sheets and do not directly hydrogen bond. The lack of a significant correlation for these pairs suggests that the β-meander and β-hairpin components of the β-barrel have a degree of structural independence. In this regard it is interesting to note that experimental studies support the ability of the β-3 meander component of the structure to fold independently of the β4–5 hairpin under some conditions [26–29].

Figure 2b shows statistics on the numbers of OB-fold domains in our dataset that have accessory secondary structures in the loops, or in the segments preceding (Nt) or following (Ct) the conserved OB-fold. All of the examples are from structures considered to be distinct domains in the SCOP and PDBsum databanks. If multiple domain proteins had been considered, the fraction of proteins with structures outside of the OB-fold would have been much larger. There is a correlation between loop length (Figure 2a) and the number of proteins within the 80-domain dataset that have non-conserved secondary structures in the respective loops (black bars in Figure 2b). The most common location of secondary structure is within the longest loop L34. Similarly, the number of residues participating in non-conserved secondary structure correlates with loop length (white bars in Figure 2b).

### 2.2. Contact densities distinguish conserved from non-conserved structures

We and others [7,17,18,30] have noted that the site-specific stability to unfolding of proteins as measured by NMR hydrogen exchange, correlates with the density of distance contacts. Thus in the OB-fold proteins we studied experimentally, the β-barrels were more resistant to unfolding [6,7,16,17] and had a larger density of Cα-Cα contacts [7]. This led us to look for similar differences between conserved and non-conserved across the entire database of OB-fold domain structures.

**Figure 3.**
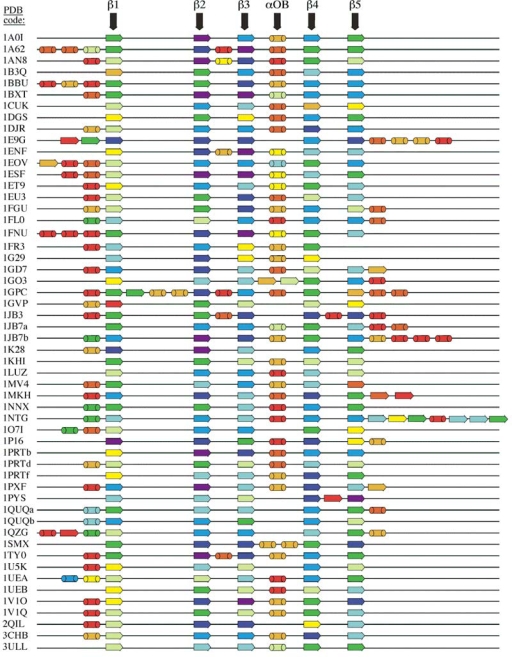
Contact densities averaged over secondary structures. PDB codes are in the left margin. Arrows and cylinders indicate β-strands and α-helices. Contact data were subject to a zero-mean normalization [31,32]. In terms of the un-normalized values, the 10-bin color scale corresponds roughly to: red, < 12; yellow, 16; dark green, 18; dark blue, 21; indigo, 24 < Cα-Cα distance contacts shorter than 10 Å per residue. The β-strands in the conserved OB-fold motif range from green to indigo. Non-conserved structures and the αOB helix range from red to yellow.

Figure 3 shows contact densities averaged over secondary structure elements in our dataset of 80 OB-fold domains. For simplicity, only the 54 domains that have secondary structures outside of the conserved β-barrel are shown, including the αOB-helix which we consider poorly conserved. A color ramp from red to indigo is used to denote increasing number of Cα-Cα distance contacts shorter than 10 Å. The five strands of the conserved β-barrel typically have the most contacts (green to indigo range). The exception is strand β1, which is structurally the most poorly conserved (yellow to blue range). The non-conserved structures outside the OB-fold have low contact densities (red to yellow range), and the poorly conserved αOB helix in loop L34 has contact densities similar to the nonconserved structures (Figure 3, Table 1).

The contact density data for each domain was subjected to normalization to a range between 0 and 1 [31,32], such that the mean occurred at 0.5 (Figure 3, Table 1). Normalization to the same mean was done because different structures have slightly different average numbers of distance contacts, depending on the method used to solve the structure (e.g. X-ray vs. NMR) and structure refinement. Similar approaches have been used to normalize crystallographic B-factors for comparisons between proteins [31,32]. The normalization slightly improved the distinction between conserved and nonconserved structure but the same trends were seen when the raw contact density data were used (Table 1). Residues in the conserved β-barrel have an average of about 19 neighboring Cα atoms within 10 Å. Non-conserved structures and the αOB helix show an average number of contacts of about 14 – 15, which approaches the value for residues outside of regular secondary structure (Table 1).

**Figure 4.**
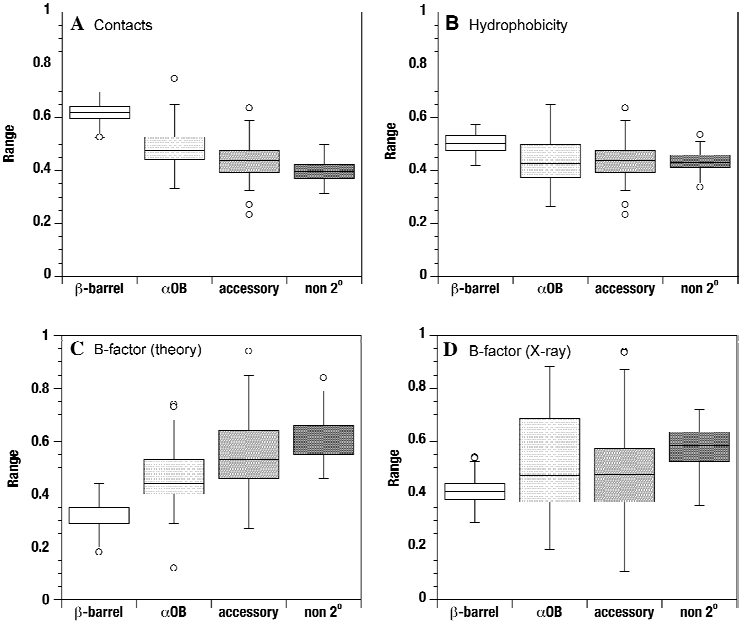
Box plots summarizing the distributions of mean values across the database of 80 distinct OB-fold domain structures. (a) Cα-Cα distance contacts shorter than 10 Å. (b) Sequence hydrophobicities calculated with the Kyte-Doolittle scale. (c) Theoretical B-factors calculated from Gaussian Network Models of the structures. (d) Crystallographic B-factors.

The values in Table 1 were calculated for individual residues. Because of the large number of residues examined, the differences in the means between conserved and non-conserved structures are highly significant (p < 0.0001). An alternative analysis is shown in the box plots of Figure 4. Here, the parameters are averaged over each of the domains in the OB-fold dataset. So for example in Figure 4A the white box describes the distributions of the average β-barrel contact densities for the 80 OB-fold domains. The mean contact densities decrease in the order conserved β-barrel > αOB helix > nonconserved secondary structure > residues not involved in secondary structure. The figure also shows that the width of the β-barrel distribution is narrower than those of the non-conserved secondary structures (Figure 4A).

### 2.3. Sequence hydrophobicity

**Figure 5.**
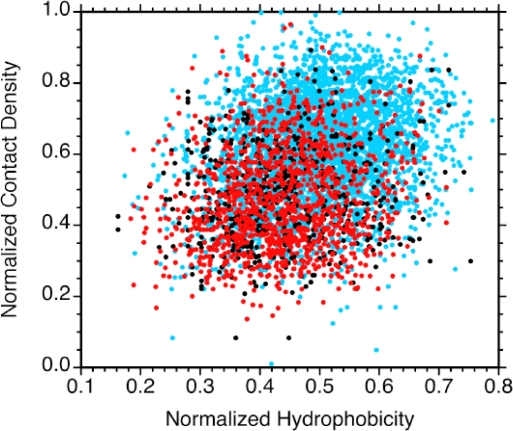
Plot of contact density versus hydrophobicity for individual residues.

Amino acid sequence hydrophobicites were calculated with a Kyte-Doolittlte scale [33] normalized to a range between 0 to 1. The hydrophobicities of the conserved β-barrels are significantly higher than the rest of the structures considered (Table 1, Figure 4B), however, the distinction is smaller than for the contact densities. Figure 5 shows a plot of contact densities versus hydrophobicities. Each symbol represents a residue in one of the 80 OB-fold domains considered. Residues from the β-barrels are in blue; residues from the αOB helices are in black, and residues from non-conserved secondary structures are in red. There is a clustering for the β-barrel residues towards the top right of the plot (blue). The residues from non-conserved secondary structure and αOB segregate to the bottom left (red and black). Hydrophobicity and contact density are significantly correlated since hydrophobic residues are more likely to be located in the interiors of proteins where they participate in more contacts with other residues. This correlation, however, is surprisingly weak with a linear correlation coefficient (R-value) of 0.28 (ρ < 0.0001 for 5,638 points). Apparently factors other than the variance in hydrophobicities, such as the details of the tertiary structures account for the majority of the variance in contact densities. Hydrophobicity is a poorer discriminator of conserved structure than contact density (Figure 4). Possibly this reflects that for soluble proteins such as the OB-fold domains the amino acid sequences have a nearly equal composition of hydrophobic and hydrophilic residues, and these are nearly randomly distributed [34]. That the conserved β-barrels have larger hydrophobicities is consistent with the location of these motifs in the cores of the structures. The hydrophobicities of the non-conserved structures including the αOB helix are statistically indistinguishable from those of residues that do not participate in regular secondary structure (Figure 4b, Table 1).

### 2.4. Theoretical B-factors

We next looked at theoretical B-factors calculated from a Gaussian Network Model of protein dynamics. The Gaussian Network Model [22–24] treats the protein as an elastic network of nodes (Cα atoms) connected by springs that correspond to inter-residue contacts within a certain distance threshold (10 Å in our case). The springs are assigned a uniform force constant, and the dynamics of the network are determined from an N x N connectivity (Kirchhoff) matrix. Residues that participate in a large subset of contacts resist changes in position and are more rigid. Diagonalization of the connectivity matrix provides information on the frequencies (eigenvalues) and modes (eigenvectors) of the correlated dynamics between residues in the elastic network [22,23]. The diagonal elements of the inverse of the connectivity matrix are proportional to the mean square fluctuations of individual residues, and can be used to calculate theoretical B-factors [22,23]. These theoretical B-factors (B_theory_) have been shown to successfully approximate crystallographic B-factors [24] and hydrogen exchange free energies [18].

**Figure 6.**
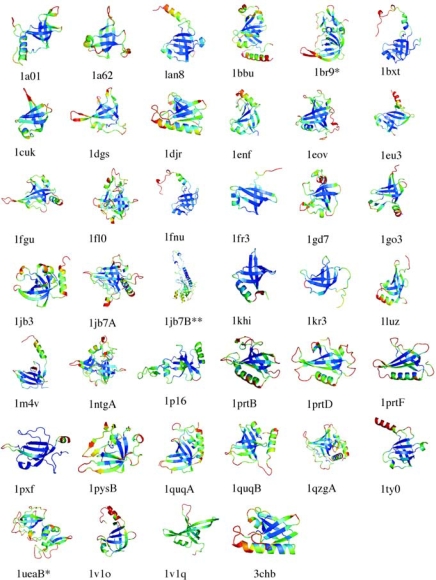
OB-fold domains color-coded according to theoretical B-factors calculated from GNM representations of the structures. Blue corresponds to rigid, red to flexible. The symbols ‘*’ and ‘**’ indicate moderate and strong exceptions from the overall trend.

Of the four parameters we considered, the B_theory_ factors from the Gaussian Network Model provided the strongest discrimination between conserved and non-conserved structures in the database of OB-fold domains (Figure 4c). In Figure 6 this is shown on a case-by-case basis for 40 of the 80 domains that were determined by X-ray crystallography and contain non-conserved secondary structure in addition to the conserved OB-fold β-barrel. Note the large diversity of structures classified to the OB-fold family. The structures are colored according to the B_theory_ factors on a scale running from blue (rigid) to red (flexible). There is a clustering of low B_theory_ values to the conserved β-barrels (Figure 6). By contrast, non-conserved motifs tend to be located at the extremities of the structures and have larger B_theory_ values suggestive of flexibility. Exceptions to this trend are denoted with one or two ‘*’ symbols after the PDB code [35] to indicate moderate and strong deviations, respectively.

The strongest exceptions are proteins that have extensive amounts of non-conserved structure comparable in size to the conserved OB-fold β-barrel (e.g. 1jb7B, 1ueaB, 1br9). These proteins start to resemble multiple domain proteins, or new folds that include the OB-motif as an ancillary component of a larger structure. Additional exceptions are seen at the edges of long β-strands that extend far from the β-barrel core (e.g. 1br9, 1cuk, 1dgs).

### 2.5. Experimental B-factors

**Figure 7.**
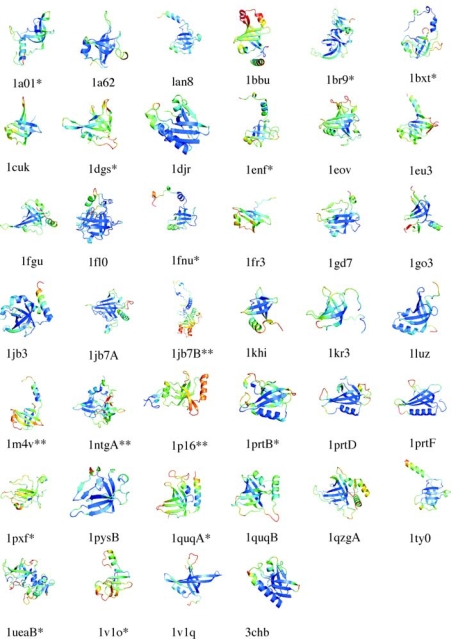
OB-fold domains color-coded according to experimental B-factors from X-ray crystallography. The coloring scheme and symbols are the same as in Figure 6.

Crystallographic B-factors are the poorest discriminators of conserved from non-conserved structures amongst the four parameters considered in this work (Figure 4d, Table 1). Nevertheless, when mapped onto the structures an overall segregation of low B-factors to the β-barrels and higher B-factors to the non-conserved structures is evident (Figure 7). The weaker trend with crystallographic B-factors reflects that these are affected both by flexibility (dynamic disorder) and by defects in crystal lattice packing (static disorder). Moreover, crystallographic B-factors for surface residues can be affected by the packing of proteins in the crystal lattice. These effects are probably averaged over the many domains analyzed. The pattern for the β-barrels to adopt lower B-factors is still seen, albeit not as pronounced as for the theoretical B-factor. One factor that may positively contribute to the trend in Figure 7 (but not Figure 6) is that the experimental B-factors tend to increase with increasing distance from the center of mass of a protein [36].

### 2.6. Differences between conserved and non-conserved structure from NMR data

We wanted to see if there were additional experimental parameters that could distinguish conserved from non-conserved motifs in OB-fold proteins. Previously we noted correlations between experimental stability data and structural parameters for the three OB-fold proteins CspA [17], LysN [16], and SN [7] that have been subject to detailed protein folding studies by NMR.

A number of other OB-fold proteins have been studied by NMR although these investigations were primarily aimed at structure determination. Consequently, data on stability and dynamics are rather limited. In our ^15^N relaxation studies of SN we noted that the consensus substrate binding loops L12, L34, and L45 (black in Figure 1) show increased flexibility by NMR relaxation measurements [37]. Loops are generally more flexible than regular secondary structure but the flexibility we observed was reduced in the presence of a substrate analogue pdTp, suggesting a functional role [37]. The flexibility of the substrate-binding loops appears to be conserved feature of non-homologous OB-fold proteins for which ^15^N relaxation data are available including CspA [38], CspB [39], LysN [16], ribosomal protein S28E [40], and the NTR domain of procollagen C-proteinase enhancer [41]. Another set of OB-fold proteins show increased flexibility in two of the three substrate binding loops based on ^15^N relaxation data: archaeal initiation factor [42], the heme chaperone Ccme [43], RNAse E S1 domain [44], N-TIMP1 [45]. The protein Cdc13-DBD has uniformly rigid backbone dynamics on the nanosecond timescale of ^1^H-^15^N NOEs but shows R2_ex_ effects characteristic of micro-millisecond motions in all three of the consensus substrate binding loops [46].

Limited hydrogen exchange (HX) data are also available for some OB-fold proteins. Typically the HX data were used to identify hydrogen-bond restraints for structure calculations rather than for quantitative studies of structural stability. For N-TIMP1 it was noted that HX protection was strong for the OB-fold β-barrel but entirely missing for two non-conserved α-helices outside the OB-fold [47]. For eukaryotic initiation factor 1A, sites within the β-barrel are protected while none of the amide protons from two α-helices outside the OB-fold survive longer than 30 min in D_2_O [48]. The HX rates for archaeal initiation factor are similar or only slightly lower for a non-conserved α-helix compared to the of the β-barrel [42]. For the telomere recognition protein Cdc13 sites protected from HX for more than 2 months were primarily distributed in the OB-fold β-barrel but some sites were also located in non-conserved structure and in a long loop between β-strands 2 and 3 [46]. We emphasize that in contrast to our studies on HX in OB-fold proteins [7] the studies described above were not quantitative, and were done in the absence of denaturant. HX can occur through a partial unfolding mechanism or through a localized breathing mechanism and the denaturant dependence of HX is needed to distinguish between the two.

In summary, NMR hydrogen exchange data for at least six types of OB-fold proteins appear to be consistent with a stability hierarchy in which the conserved β-barrel motif is the most resistant to unfolding but there is a need to substantiate this conclusion with more systematic and quantitative studies. Information on stability and dynamics may prove as valuable in discerning evolutionary relationships amongst proteins as data on protein amino acid sequences and three-dimensional structures.

## 3. Discussion

Our results indicate that the conserved β-barrel motifs of OB-fold domains are richer in hydrophobic residues and have larger numbers of inter-residue contacts than the non-conserved structures. The conserved motifs also tend to have smaller experimental and theoretical B-factors (Table 1) suggestive of decreased flexibility. The four parameters we examined are interdependent. For globular proteins like the OB-fold domains, sites with the highest contact densities will be located in the hydrophobic cores of the structures. Mutations of core residues would require rearrangement of a larger network of contacts, and would be more likely to give rise to non-functional proteins than substitutions at the hydrophilic periphery. For the same reasons core sites of proteins are likely to be less dynamic and more stable to unfolding. It is important to note that we are comparing relative stabilities within protein structures. The OB-fold family displays a large range of global stabilities to unfolding (e.g. 2.8 kcal/mol for CspA, 6.2 kcal/mol for LysN), and stabilities vary considerably between mesophilic and thermophilic variants of the same protein such as the cold shock proteins [49], or between single-site mutants [19].

At this time we do not know if the differences between conserved and non-conserved structures found in this study represent a general feature of globular protein folds. An important control is to rule out an intrinsic bias in contact densities between α-helices and β-sheets. This could occur because of intrinsic geometric differences between the two types of structures such as the arrangements of Cα atoms in approximately flat planes in β-sheets and around a cylinder in an α-helix. As previously mentioned the parameters we looked at are interrelated, so that a bias in contact density for a given type of secondary structure could also affect other parameters such as the theoretical B-factors. To investigate the possibility of bias in contact densities between α-helix and β-sheet structures we looked at 30 high-quality X-ray structures of proteins with 10 each from all-α folds, all-β folds, and folds containing both α-helices and β-sheets (detailed in the Experimental section). We compiled un-normalized statistics and found that with our 10 Å Cα-Cα distance threshold the average number of contacts was 19.6 ± 4.8 for 1,017 residues in β-sheet structures compared to 17.5 ± 4.9 for 1,273 residues in α-helix structures. For comparison, Table 1 shows the corresponding mean contact values are 18.8 ± 4.6 for 3,938 residues in the conserved β-barrel and 14.7 ± 5.0 for 1,369 residues in nonconserved structures in the OB-fold domains. Thus while the controls structures show that β-sheet residues tend to be involved in a larger number of contacts than those in α-helices, the differences seen between conserved and non-conserved structures in OB-fold proteins are at least twice as large. We point out that while α-helices make up most of the non-conserved secondary structures, there are some examples of OB-fold proteins that contain β-sheet structure and that these usually have lower contact densities than the conserved β-barrels (Figure 3). Moreover we are unaware of any literature reports that would indicate a systematically larger stability in partially folded states for β-sheets compared to α-helices. Taken together these observations suggest that the differences in structural parameters between conserved and non-conserved components of the OB-fold are due to the folding hierarchy of the structures. Whether these properties are a general feature remains to be seen, and will require extending the types of analyses described here to other types of folds.

Our results appear to be consistent with members of the OB-fold superfamily having originated from an ancestral β-barrel motif that diversified its function by developing novel structural features. Structural augmentations could have been used to supplement specificity onto a rudimentary oligomer-binding function - for example to distinguish between tRNA and DNA oligonucleotides. The alternative convergent mechanism, where the β-barrel motif could have become incorporated into a pre-existing structure to play a functional or structural role seems less likely given that the β-barrel usually corresponds to the main component of the structures. It is nevertheless not possible to rule out the possibility of convergent evolution in particular instances. The flexibility of the substrate binding loops suggested by NMR ^15^N relaxation studies, for example seems consistent with a convergent role. Although the conclusion that the OB-fold domains derived from an initially simple β-barrel motif that captured additional structural elements is attractive, the alternative of a complex motif that lost structural elements is also plausible in light of the fact that many of the OB-fold domains have the αOB α-helix between strands 3 and 4 missing (Figure 3).

The modular arrangement of structure seen for OB-fold proteins could result in partially folded states. These would be strongly selected against during evolution since they could lead to alternative structures and to protein misfolding. This selective pressure, however, may need to be balanced against the requirement of minimizing the disruption to pre-existing structure. The reconciliation between these opposing forces when incomplete, could account for the stability differences between conserved and non-conserved structures. Support for this idea comes from the observation that the modularity of structure as determined by contact densities or B-factors starts to break down when the conserved OB-fold β-barrel is no longer the main part of the structure. In these cases the complement of the structure can afford a sufficiently large hydrophobic core to ensure the viability of the protein even when the OB-fold part of the structure is substantially altered. Eventually, the changes in the OB-fold module could become so extensive and the motif so integrated into the overall folding topology that the resulting structure is more properly classified as a separate fold. The balance between the need to develop new structural features and the need to maintain stable structural foundations could have resulted in the punctuation of the protein structure continuum with recurrent motifs that we have come to recognize as ‘protein folds’.

## 4. Conclusions

The work presented shows that the structures of OB-fold proteins are arranged in a hierarchic fashion. The modularity of structure is apparent in the distinct structure and sequence properties of the β-barrel when it coexists with other secondary structures as part of a domain (Figures 3–7). The experimentally observed differences in microscopic stabilities of conserved and non-conserved structures in OB-fold proteins correlate with differences in sequence and structure properties. The nonconserved structures have slightly more polar sequences, smaller numbers of neighboring residues in the structure, and larger crystallographic B-factors. The observations suggest a structure evolution mechanism in which novel features develop at the periphery, while hydrophobic cores are conserved.

## 5. Experimental Section

### 5.1. OB-fold database selection

The database of 80 unique OB-fold domains was created using the September 2007 release of the SCOP fold classification [3,21]. SCOP classifies protein structures according to a descending hierarchy of class > fold > superfamily > family > protein domain > species. We chose “protein domain” as the unit of classification because this is also the basis of the SCOP taxonomy [21], and because proteins within the same “family” can differ in the number of non-conserved accessory structures. For example, the anticodon-binding domains of lysyl (PDB 1KRS) and aspartyl (PDB 1KRS) tRNA synthetases share ~ 15% sequence homology and have different numbers of accessory α-helices, even though the proteins are assigned to the same family. We therefore felt that a classification at the domain level would be most suitable for sampling differences between conserved and nonconserved structure elements - the aim of the investigation. When multiple structures were available for a domain we chose the first entry listed in SCOP.

Of the 95 domains classified as OB-fold motifs in SCOP, six structures (1CKM, 1HR0, 1J5E_L, 1J5E_Q, 1MIU, 1X9N) were discarded because of low resolution and/or errors, including two structures that had a knot in the main-chain (1X9N and 1CKM). These domains were usually part of very large complexes such as ribosomes or nucleosomes and all but 1CKM had crystallographic resolutions ≥ 3 Å. Another nine structures (1H9M, 1K0S, 1NY4, 1H9S, 1OXX, 1RL2, 1KLU, 1TWF, 1V43) were discarded because they are probably not OB-fold structures. Thus 1RL2 has only the three-stranded β-meander component of the OB-fold, 1V43 and 1NY4 have only four of the five β-strands, and 1TWF has six strands. 1OXX, 1K0S, 1H9M and 1H9S have complicated topologies that can be related to the OB-fold through domain duplication and interdigitation to give structures with as many as ten β-strands. There may be subjective reasons based on the functions of the proteins to include them as OB-fold domains, even though structurally they represent rather large excursions. The OB-fold is structurally more similar to the SH3 fold than to the putative duplicated OB-fold topologies. We excluded these structures from our database in order to avoid the well-known Russian Doll effect [50] where complex structures can be subdivided into simpler structural motifs in the absence of a biological or evolutionary relationship. Exclusion of the 15 structures above left us with 80 OB-fold domains for our analyses.

### 5.2. Secondary structure assignment

Secondary structures were assigned using the PDBsum server [51] using the topology diagrams available for each domain. The topology diagrams give the sequence positions of each secondary structure element, and also demarcate domain boundaries in multi-domain or oligomeric structures. In addition, we individually checked the secondary structure assignments for each domain using the cartoon diagram and sequence mode utilities of the PyMol program [52]. If there were conflicts between the two methods, we chose the assignments obtained by visual inspection since the latter takes into account the placement of secondary structures within the context of the OB-fold topology. One problem arises with the first strand of the conserved OB-fold motif, β1. The β1 strand has a conserved bulge that allows its N-terminus to pair with β4, and its C-terminus to pair with β2 (Figure 1). In some of the structures the break in the strand is extensive and the secondary structure is strictly two β-strands separated by an irregular linker. Less frequently these types of breaks also occur with other strands such as β4 and β5. To allow comparisons between structures we focused on the topological properties of strand β1, defining it as a continuous uninterrupted strand rather than restricting our analysis to only those residues with φ,ψ dihedral angles in the β-sheet region of conformational space. This approach may over-estimate the flexibility of strand β1 and of the β-barrel, because of the inclusion of residues in the bulge/linker region that are not part of regular secondary structure. While we do see a higher flexibility for strand β1 compared to the other strands, it remains much more rigid than either the OB-fold α-helix or the non-conserved secondary structures (Table 1). The complete list of OB-fold domains in our database together with secondary structure assignments for each domain can be obtained by request from the corresponding author.

### 5.3. Analysis of sequence and structure parameters

The PDB file for each OB-fold domain in our database was run through the oGNM server [23] to calculate three structural parameters: the number of distance contacts, experimental B-factors, and theoretical B-factors. Whenever necessary we used PDB files edited to contain only the OB-fold portion of multi-domain structures. For calculating the number of Cα-Cα contacts and theoretical B-factors we used a distance cutoff of 10 Å.

To test whether residues in β-sheets have systematically larger number of Cα-Cα distance contacts than residues in α-helical structure we built a small control database. We first chose all structures in the PDB with resolutions better than 1 Å and R-factors lower than 0.15, for proteins that had between 50 and 250 amino acids. These structures were run through the PDB select server [53] to remove redundant homologues at the 25% sequence identity threshold. Of the resulting 80 structures we selected the top 10 with predominantly α-helical structure (PDB-codes: 1L9L, 207A, 1MN8_D, 1OAI, 2NRL, 2PVB, 1C75A, 1XG0_C, 1MC2, 1XMK); the top 10 all-β structure (PDB-codes: 1F94, 2CHH, 1OD3, 1OK0, 2GUD, 2BT9_B, 209S, 1W0N, 1ZUU, 2RH2); and the 10 with the ratio of residues in α-helix and β-sheet structure closest to 1 (PDB-codes: 2JFR, 2I4A; 1F9Y, 1MWQ 2QSJA, 2NWZ, 1XG0_B, 2GGC, 1IUA, 3DHA).

Amino acid sequences for OB-fold domains were collated from their respective PDB entries [35]. Domain information from PDBsum [51] together with the sequence mode utility of the PyMol program [52] were used to determine the start and end sequence positions of OB-fold domains that were parts of larger structures. The sequences were submitted to the ProtScale server [54] and Kyte-Doolittle hydrophobicities [33] were calculated using a default smoothing window of nine residues.

Once all four parameters were compiled the data were entered into a spreadsheet and a flag (0 or 1) was used to tag each residue to indicate its participation in a given secondary structure (e.g. strand β3). Sorting of the data according to the flags was used to extract the structure and sequence parameters of different secondary structure types.

## Figures and Tables

**Table 1.** Averages of parameters that distinguish conserved and non-conserved structures in the OB-fold superfamily*^a^*.

**2° Structure**	**# Residues ^b^**	**# Contacts (raw)**	**# Contacts (normalized)**	**Theory B-factor**	**X-ray B-factor**	**Hydrophobicity**
β-barrel	3,938	18.8 ± 4.6	0.62 ± 0.13	0.32 ± 0.16	0.41 ± 0.17	0.50 ± 0.09
strand β1	1,085	17.8 ± 4.8	0.59 ± 0.15	0.34 ± 0.16	0.44 ± 0.18	0.49 ± 0.10
strand β2	720	19.7 ± 4.6	0.64 ± 0.15	0.31 ± 0.17	0.41 ± 0.16	0.51 ± 0.09
strand β3	614	19.7 ± 4.5	0.64 ± 0.14	0.30 ± 0.14	0.39 ± 0.15	0.50 ± 0.08
strand β4	804	19.0 ± 4.2	0.62 ± 0.13	0.31 ± 0.15	0.40 ± 0.16	0.50 ± 0.09
strand β5	715	18.7 ± 4.4	0.61 ± 0.15	0.32 ± 0.17	0.39 ± 0.17	0.49 ± 0.09
βOB	588	14.4 ± 3.9	0.48 ± 0.13	0.46 ± 0.16	0.52 ± 0.21	0.44 ± 0.10
accessory 2°	1,369	14.7 ± 5.0	0.46 ± 0.15	0.54 ± 0.26	0.49 ± 0.22	0.44 ± 0.10
non-2°	5,023	12.5 ± 5.0	0.42 ± 0.17	0.57 ± 0.27	0.56 ± 0.24	0.43 ± 0.10

*^a^* The data are presented as means ± 1 standard deviation. For each variable except the raw number of contacts, values were normalized between 0 (low) and 1 (high) such that the overall mean is at 0.5 [31, 32].

*^b^*Number of residues is for the contact density parameter. For other parameters this number may be slightly reduced, for example NMR structures will not have B-factors.
